# News and Events of the Week

**Published:** 1910-06-11

**Authors:** 


### News and Events of the Week.

Mr. J. F. Harrington, of Kensington, has been re-elected President of the Council of the Pharmaceutical Society; Mr. W. L. Currie, of Glasgow, Vice-President; and Mr. W. H. Gibson, of Brighton, Treasurer.

The managing committee of the Sleeping Sickness Bureau has received from the Agent-General for the Transvaal £500 as a contribution to the funds of the bureau, made by the Transvaal Government.

Dr. Wolferstan Thomas, assistant lecturer in the Liverpool School of Tropical Medicine, has been appointed director of the new laboratories at Manaos in the State of Amazonas, Brazil. These new laboratories are supported entirely by foreign business firms in Manaos. Dr. Thomas has also been commissioned to proceed to Porto Velho, on the Madeira river, to investigate diseases endemic to that district which tend to retard its commercial progress.

The Vice-Chancellor of Cambridge University has published to the Senate a minute of the Council of Trinity College, urging the need of a Professorship of Bio-Chemistry, and stating that, to increase the provision for higher teaching and research in that subject, pending the establishment of a professorship, the College has appointed Mr. F. G. Hopkins, at present Reader in Chemical Physiology, to a Praelectorship in Bio-Chemistry, and proposes to elect him to a Fellowship.

A special meeting of Fellows of the Royal College of Surgeons will take place at the College on July 7 to elect four Fellows to fill the vacancies occasioned by the retirement in rotation of Mr. John Hammond Morgan and Mr. Charles Barrett Lockwood, by the death of Mr. Henry Hugh Clutton, and by the resignation of Mr. George Arthur Wright, of Manchester. It is understood that Mr. Lockwood, of St. Bartholomew's Hospital, will seek re-election, and that Mr. Charles Alfred Ballance, M.V.O., of St. Thomas's Hospital; Mr. John Bland Sutton, of Middlesex Hospital; Mr. James Ernest Lane, of St. Mary's Hospital; and Mr. Bilton Pollard, of University College Hospital, will be candidates for election.

The Council of the Midwives Institute, on behalf of the various interests which it represents, has sent to the Lord President of the Council a petition to amend certain clauses in the Midwives Bill now before the House of Lords. The principal alterations asked for are: (1) Two representatives on the Central Midwives Board instead of one; (2) the omission of Clause 11, Section 1, which makes it compulsory on any midwife acting as a nurse at a maternity case to notify the birth should the doctor not be present at the time; (3) that grants in aid of maintenance as well as training should be made by local supervising authorities in districts where a midwife is unable to make a living owing to the sparseness of the population; (4) that the payment of medical practitioners called to assist midwives should be undertaken by the local supervising authorities instead of the Poor Law Guardians, thus avoiding the stigma which always attaches to any Poor Law help, and which, in the case of accidents in childbirth, is totally uncalled for.

The Council of Sheffield University has accepted, with thanks, an offer from the staff of the Children's Hospital to afford teaching facilities at the hospital to University medical students.

The expedition of medical men organised by Sir Rubert Boyce sailed from Liverpool last week to deal with the outbreak of yellow fever on the West Coast of Africa. With the exception of Sir Rubert Boyce all were trained in the London School of Tropical Medicine and volunteered their services to the Government.

The 1910 edition of "Printers' Pie" is as good as ever, and perhaps no other periodical (even including the "English Review") manages to collect such a motley of talent, both pictorial and literary, as this festival souvenir. Mr. Hassall again leads the company of illustrators with a cover design and other coloured drawings in his well-known manner. William de Morgan, Barry Pain, and Robert Hitchens are three names gathered at random from the literary contributors. There is a great deal of fun and pleasure in this volume for the modest shilling which it costs.

A case of leprosy in the Whitechapel Union Infirmary is causing the Guardians some anxiety. The case, after being in a hospital at Oxford, was transferred *via* the London Hospital to Whitechapel, and is the first which the clerk of the Guardians recollects as having come under the control of his board. It is not a bad case from the point of view of infection, but there has been difficulty with regard to isolation, and inquiries are being made of the Metropolitan Asylums Board as to where the patient can be sent, there being no provision for the treatment of lepers owing to their rarity.

Miss Martha Edwards has recently died at the Royal Hospital for Incurables, Putney Heath, after being an in-patient for 48 years. Although confined to her bed for the last quarter of a century, Miss Edwards maintained a bright and cheerful disposition. Miss Sarah Stanford, another of the in-patients of the Royal Hospital for Incurables, Putney Heath, who is 84 years of age, is still alive, and has been longer in the institution than any other patient. She was admitted to the Royal Hospital for Incurables so long ago as the year 1858, and has therefore been an inmate of this charity for 52 years. Miss Stanford is blind and paralysed, but she is still able to do a little knitting daily.

Sir James Crichton-Browne received last week the freedom of Dumfries, his native city. The ceremony took place in the reconstructed Town Hall, which he opened. In returning thanks, Sir James recalled that eight years ago the same honour was conferred on his son, who was one of a group of soldiers returned from the South African war; and said it was a great honour to be admitted to the Valhalla they had created. In opening the Town Hall, Sir James said that he liked a little extravagance in town halls, so that they might appeal to the imagination and impress all beholders with a sense of beauty, fitness, and solidarity. He would like to see a real Mansion House in Dumfries, a stately pile in the best Scottish baronial style.

Mr. H. S. Pendlebury, F.R.C.S., has been appointed assistant examiner in surgery to the Apothecaries' Society of London, in place of Mr. Stonham, who has retired by rotation.

The Committee in charge of the presentation portraits of Professor Ogston request intending contributors to forward their subscriptions at an early date to the Honorary Secretary, Dr. Mackenzie Booth, 1 Carden Place, Aberdeen.

Among the members of the newly appointed first Union Ministry in South Africa is Dr. Charles O'Grady Gubbins, who becomes Minister without portfolio. Dr. Gubbins qualified M.B., B.Ch., at Dublin in 1878, and went out to Natal early in the eighties. He was in practice at Newcastle, and was a member of the late Natal Legislative Assembly. His inclusion in the Ministry will be welcomed by medical men in South Africa, as he has always shown a deep interest in matters which affect the welfare of the profession.

The annual meeting of the National Society of Day Nurseries was held on Monday last, at 35 Chesham Place, S.W., by kind permission of Mr. and the Baroness de Goldsmid. The annual report for the past year showed that much progress had been made in all parts of the country in the work of establishing new crèches and of improving those already in existence. Twenty-six day nurseries had become affiliated to the Society, and were in receipt of the annual grants and certificates signed by the royal president. Much educative work had been carried on by the Society by means of printed matter, and also by practical demonstrations of model crèches at exhibitions, such as those at Olympia, Glasgow, and the recent Ideal Home Exhibition. Thanks in great measure to the receipt of a legacy of £500 from the late Louisa Lady Goldsmid, the Society had now £1,300 invested. This investment they hoped to retain in order to use the income towards the payment of working expenses. An appeal was made for further contributions to assist the Society in its ever widening operations. In moving the adoption of the report, Lady Helmsley, who occupied the chair, made a touching appeal for assistance in checking the present deplorable waste of infant life and in making proper provision for the little ones whose mothers were compelled to go out to daily work. To this intent, not only were day nurseries all over the kingdom inspected and supported and their managers advised, but the opportunity was taken of instructing the mothers in the rearing of their children, and of giving proper training to nursemaids. These were objects which should appeal to all classes of the community. Applications were constantly being made for the Society's assistance, and steps were now being taken to see that all the affiliated crèches were being afforded proper protection against fire. The power of the Society to respond to these appeals depended entirely on the support it received from the general public. The Society could not be too grateful for the personal interest graciously taken in its work by the Princess Christian. Mr. Hoffnung-Goldsmid, the Hon. Treasurer, seconded the adoption of the report, which was carried unanimously. Dr. F. S. Toogood, Chairman of the Executive Committee, at the conclusion of the business, in moving a vote of thanks to the Chairman, said that the Society's indebtedness to Lady Helmsley for her devotion to its work could not be overstated. The vote was carried with acclamation. Contributions, especially annual subscriptions, are earnestly solicited, and should be sent to the Hon. Treasurer, Mr. Hoffnung-Goldsmid, or to the Secretary, Mr. Herbert H. Jennings, at the office of the Society, 1 Sydney Street, Fulham Road, London, S.W.

